# A genome-wide analysis of DNA methylation identifies a novel association signal for Lp(a) concentrations in the *LPA* promoter

**DOI:** 10.1371/journal.pone.0232073

**Published:** 2020-04-28

**Authors:** Stefan Coassin, Natascha Hermann-Kleiter, Margot Haun, Simone Wahl, Rory Wilson, Bernhard Paulweber, Sonja Kunze, Thomas Meitinger, Konstantin Strauch, Annette Peters, Melanie Waldenberger, Florian Kronenberg, Claudia Lamina

**Affiliations:** 1 Department of Genetics and Pharmacology, Institute of Genetic Epidemiology, Medical University of Innsbruck, Innsbruck, Austria; 2 Department of Genetics and Pharmacology, Institute of Cell Genetics, Medical University of Innsbruck, Innsbruck, Austria; 3 Research Unit of Molecular Epidemiology, Helmholtz Zentrum München–German Research Center for Environmental Health, Neuherberg, Germany; 4 Institute of Epidemiology II, Helmholtz Zentrum München–German Research Center for Environmental Health, Neuherberg, Germany; 5 First Department of Internal Medicine, Paracelsus Private Medical University, Salzburg, Austria; 6 German Center for Cardiovascular Research (DZHK), Partner Site Munich Heart Alliance, Munich, Germany; 7 Institute of Human Genetics, Technische Universität München, Munich, Germany; 8 German Research Center for Environmental Health, Institute of Human Genetics, Helmholtz Zentrum München, Neuherberg, Germany; 9 German Research Center for Environmental Health, Institute of Genetic Epidemiology, Helmholtz Zentrum München, Neuherberg, Germany; 10 Institute of Medical Informatics, Biometry, and Epidemiology, Ludwig-Maximilians-Universität, Munich, Germany; 11 Institute of Medical Biostatistics, Epidemiology and Informatics (IMBEI), University Medical Center, Johannes Gutenberg University, Mainz, Germany; Case Western Reserve University, UNITED STATES

## Abstract

Lipoprotein(a) [Lp(a)] is a major cardiovascular risk factor, which is largely genetically determined by one major gene locus, the *LPA* gene. Many aspects of the transcriptional regulation of *LPA* are poorly understood and the role of epigenetics has not been addressed yet. Therefore, we conducted an epigenome-wide analysis of DNA methylation on Lp(a) levels in two population-based studies (total n = 2208). We identified a CpG site in the *LPA* promoter which was significantly associated with Lp(a) concentrations. Surprisingly, the identified CpG site was found to overlap the SNP rs76735376. We genotyped this SNP de-novo in three studies (total n = 7512). The minor allele of rs76735376 (1.1% minor allele frequency) was associated with increased Lp(a) values (p = 1.01e-59) and explained 3.5% of the variation of Lp(a). Statistical mediation analysis showed that the effect on Lp(a) is rather originating from the base change itself and is not mediated by DNA methylation levels. This finding is supported by eQTL data from 208 liver tissue samples from the GTEx project, which shows a significant association of the rs76735376 minor allele with increased *LPA* expression. To evaluate, whether the association signal at rs76735376 may actually be derived from a stronger eQTL signal in LD with this SNP, eQTL association results of all correlated SNPs (r^2^≥0.1) were integrated with genetic association results. This analysis pinpointed to rs10455872 as the potential trigger of the effect of rs76735376. Furthermore, both SNPs coincide with short apo(a) isoforms. Adjusting for both, rs10455872 and the apo(a) isoforms diminished the effect size of rs76735376 to 5.38 mg/dL (p = 0.0463). This indicates that the effect of rs76735376 can be explained by both an independent effect of the SNP and a strong correlation with rs10455872 and apo(a) isoforms.

## Introduction

High Lipoprotein(a) [Lp(a)] concentrations represent a major risk factor for several cardiovascular diseases that is widely present in the population [1–3]. Depending on the cut-off used to define the normal range (30 mg/dL (reviewed in [4]) or 50 mg/dL[5]), 15% to 25% percent of the Caucasian population present Lp(a) concentrations that put them at increased cardiovascular risk[1–3]. Increased Lp(a) concentrations are consistently associated with cardiovascular diseases such as coronary artery disease[6,7] and myocardial infarction[8], stroke[9], peripheral artery disease[7,10], calcified aortic valve stenosis [7,11,12] and heart failure[13] and even cardiovascular as well as total mortality[7,14]. Conversely, very low Lp(a) concentrations have been shown to be associated with type 2 diabetes[7,15]. The associations with cardiovascular phenotypes as well as the unexpected association with diabetes[15] have been supported by several Mendelian randomization studies [6,12,13,16–19], underscoring an likely causal impact of Lp(a) on these phenotypes.

Lp(a) concentrations are mostly genetically determined by one major gene locus, the *LPA* gene[20], which presents a peculiar structure consisting of several so-called kringle domains (kringle 4 type one to type 10 and kringle 5; abbreviated KIV-1 to -10 and KV). The KIV-2 domain (consisting of two exons) is encoded by a hypervariable copy number variation, which can be present in 1 to >40 copies per allele[1] and thus generates >40 isoforms of the protein [apolipoprotein(a)]. In Caucasians, low molecular weight isoforms (11–22 KIV repeats) are associated with 4–5 times higher Lp(a) than high molecular isoforms with >22 repeats.

The apo(a) isoform determined by the KIV-2 repeat number is the strongest contributor to Lp(a) concentration variance and explains ≈30–70% of the total trait variance, with the complete *LPA* locus explaining >90%[7,20,21], Interestingly, Lp(a) levels of the same-sized isoforms vary by 200-fold in the general population[1] but <2.5-fold within families[22], suggesting the existence of genetic variation strongly modifying the impact of the isoform. Indeed, genome-wide SNP-bound heritability has been recently estimated to be ≈49% in a German population[23].

Some high impact loss-of-function variants have been described[24–28] but regulatory variants have been elusive so far. The search for regulatory variants has so far focused on the promoter region[29–31] and a known enhancer region located 15 to 25 kb upstream of *LPA*[32], as well as on genome-wide association study efforts[23,33,34]. Three variants in the enhancer region have been found to regulate *LPA* expression in reporter assays[32]. Conversely, the promoter region of *LPA* is less well defined. Depending on whether a reference transcript with a leading non-coding first exon[29] (UCSC Genome browser hg38 annotation) or without it[30,31] (NCBI NM_005577.2) was used, two different genomic regions located ≈4 kb apart from each other have been investigated as putative promoters. The most extensively studied region extends about 1.4 kb upstream of the translation start. Some regulatory variants[35,36] and liver-specific regulatory elements[37] have been described in this region, as well as a pentanucleotide repeat (PNR) polymorphism with 5–12 repeats[30]. The latter is significantly associated with Lp(a) levels in Caucasians[38,39]. Early reports indicated a causal impact on promoter activity[30], which was not replicated in later studies[31]. Since the association of the PNR with Lp(a) is, however, well replicated[38,39], it is likely that the PNR is simply in linkage disequilibrium with a regulatory variant[39].

Epigenetic regulation presents an additional layer of genetic regulation acting via chemical modifications of either histones[40] or the DNA sequence itself[41]. DNA methylation is a process by which methyl groups are added to the DNA molecule[41]. 5-Methylcytosine can be activating, when occurring in gene bodies, or repressing, when occurring in promoters[41] (albeit the repressing action of promoter methylation extends into the 5’ region of the gene body[42]). DNA methylation may act by modifying the binding affinity of transcription factors[43] or recruiting silencing factors causing heterochromatinization[41]. Variation in the methylation level can thus affect both binding affinity of transcription factors and chromatin organization.

Allele-specific methylation (ASM) at SNPs represents a special case of gene regulation via methylation. Up to 50,000, mostly cis-acting, ASM-SNPs have been reported[42,44] so far. The large majority was located in CpG dinucleotides[45], where the SNP either creates or abolishes a CpG site (CpG-SNP). Such ASM-SNPs have been reported for e.g. inflammatory bowel disease[46], osteoarthritis susceptibility[47], atopic dermatitis[48], triglyceride levels[49] and diabetes[50]. Even methylation close to a SNP site may already modulate the allele specific binding of transcription factors to the SNP site itself[51].

The impact of methylation on regulation of lipid phenotypes has been studied to a lesser extent than germline SNPs on these phenotypes. The studies performed so far reported clear associations with lipid phenotypes such as VLDL-C, HDL-C, LDL-C, triglycerides and total cholesterol and even lipid-related diseases (reviewed in[52]), but none of these studies has addressed Lp(a). Therefore we report here the first epigenome-wide methylation association study on Lp(a) concentrations.

## Results

### Methylation analysis and methylation association study

Descriptive statistics of the participating studies are given in S3 Table. One CpG site (cg17028067) located in the promoter region of the *LPA* gene was significantly associated with Lp(a) in the discovery sample KORA F4 (p = 6.04e-11, Table 1). This site was replicated in KORA F3 with direction-consistent effect size (p = 0.0001 in KORA F3, meta-analysis results: p = 2.61e-14; Table 1). This association result did not change after additionally adjusting for LDL-C in KORA F4 (β(se) = -7.49(0.93), p = 5.11e-11).

**Table 1:** Results of the linear model of cg site cg17028067 on Lp(a).

	Study	n	Beta	Se	p-value
Model 1*	KORA F4	1724	-7.58	0.94	6.04e-11
	KORA F3	484	-11.08	1.91	0.0001
	**Meta-analysis**	**2208**	**-8.26**	**0.84**	**2.61e-14**
Model 2^†^ (adjusted for the smaller of both apo(a) isoforms)	KORA F4	1710	-4.66	0.82	7.24e-05
	KORA F3	477	-8.72	1.67	0.0007
	**Meta-analysis**	**2187**	**-5.45**	**0.74**	**4.196e-07**
Model 1* (in the subgroup of participants with one isoform equal to 18, 19 or 20 KIV repeats)	KORA F4	223	-3.17	1.72	0.00265

The p-value is derived from the log-transformed model, whereas beta estimate and standard error (se) are derived from the model on the original scale of Lp(a) to ease interpretability and refer to a change of 0.1 in methylation beta-value.

*Model 1: adjusted for age, sex, Houseman variables [87], 20 principal components of the control probes

^†^Model 2: as Model 1 + additionally adjusted for isoforms

Since low methylation beta-values of cg17028067 (mean ± sd in KORA F4 = 0.811 ± 0.068; in KORA F3: 0.782 ± 0.061) were occurring predominantly in low molecular weight apo(a) isoforms with 18 to 20 KIV repeats (S4 Fig), the analysis was extended as follows (Table 1): first additionally adjusting for the lower of both apo(a) isoforms and secondly analyzing only the subgroup of participants with one isoform equal to 18–20 repeats (only in KORA F4 due to low sample size in KORA F3). Although the association was partially attenuated after adjustment for apo(a) isoforms (p = 4.20e-7, n = 2187) and in the subgroup of isoforms 18–20 (p = 0.00265, n = 223), it still remained highly significant, indicating that the association is not only a consequence of a correlation of the SNP with small apo(a) isoforms.

### SNP genotyping

**Fig 1:**
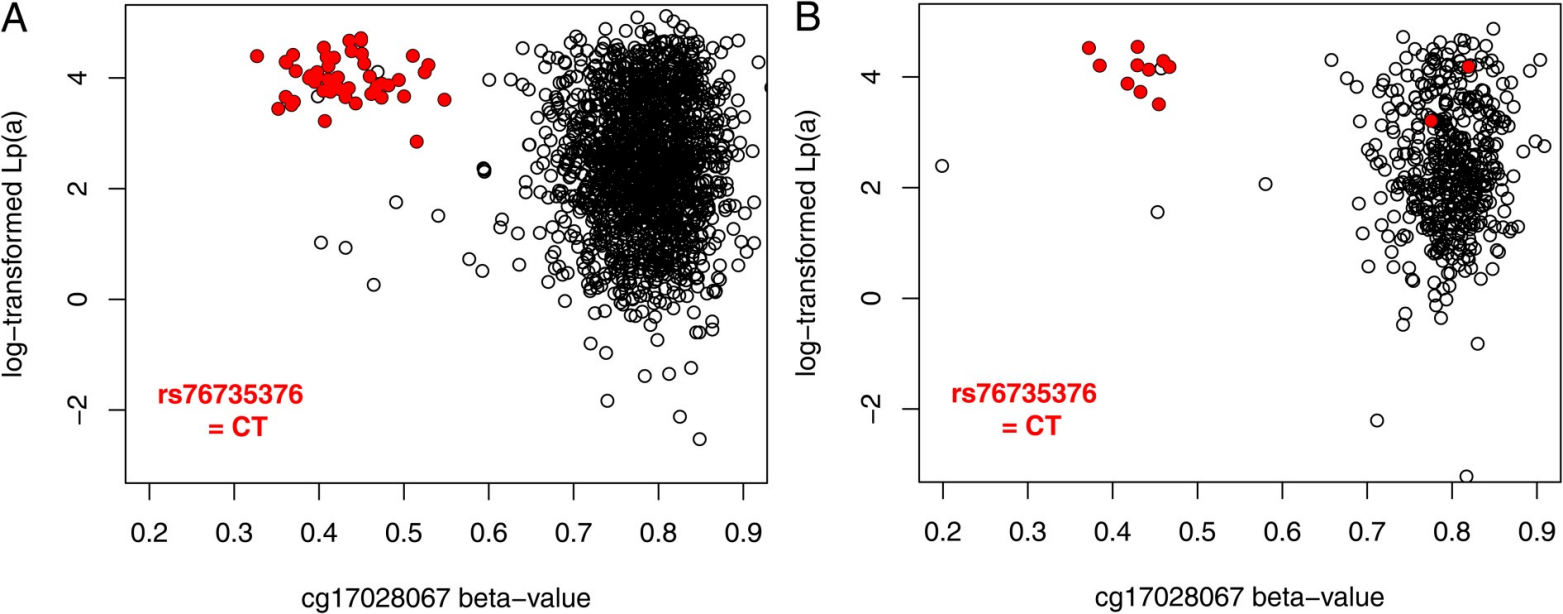
Association of methylation levels with Lp(a) values. Relationship of methylation level at cg17028067 (x axis), Lp(a) values (log-transformed (y axis) and rs76735376 genotype in the KORA F4 (panel A, n = 1724) and KORA F3 study (panel B, n = 484). Heterozygous carriers of rs76735376 (n = 49 in KORA F4, n = 12 in KORA F3) show roughly halved methylation levels and higher Lp(a) levels with less variance.

Surprisingly, the identified cg site was found to overlap a SNP (rs76735376). This C>T (reverse strand, i.e. transcribed strand of *LPA*) polymorphism is located exactly at the cytosine base of the CpG site cg17028067. The minor allele thus abolishes one potential methylation site by replacing the cytosine base of the CpG by a thymidine, thus effectively reducing the amount of available methylation target sites (Fig 1). This SNP had not been genotyped directly with the earlier SNP microarray but had been imputed with a low imputation quality (imputation quality info = 0.4) and a reported very low minor allele frequency of ≈0.1%. Therefore, this cg site had not been removed from the primary analysis dataset when SNP positions were masked. Even more surprising, subsequent de novo genotyping and confirmatory Sanger sequencing in KORA F3, KORA F4 and SAPHIR revealed that this SNP was considerably more frequent than reported in the imputed data with a true MAF of 1.1% and a carrier frequency of 2.2% (S4 Table).

### Validation by genotyping and bisulfite sequencing

The methylation of the region was validated by bisulfite sequencing in 8 samples of the SAPHIR study (4 homozygotes for the major allele of rs76735376 and 4 heterozygotes, S5A Fig). Besides cg17028067 nine additional CpG sites nearby cg17028067 were all nearly 100% methylated (S2 Fig). None of these sites were represented on the HumanMethylation450 BeadChip array. The methylation level of the cg sites nearby was independent from the status of cg17028067. This indicates that the whole region might contain CpG dinucleotides, which are target of DNA methylation and can thus potentially affect *LPA* expression. In heterozygous carriers of the SNP rs76735376 the methylation at cg17028067 was reduced by 50% as would be expected (S5B Fig).

### SNP association analysis

**Fig 2:**
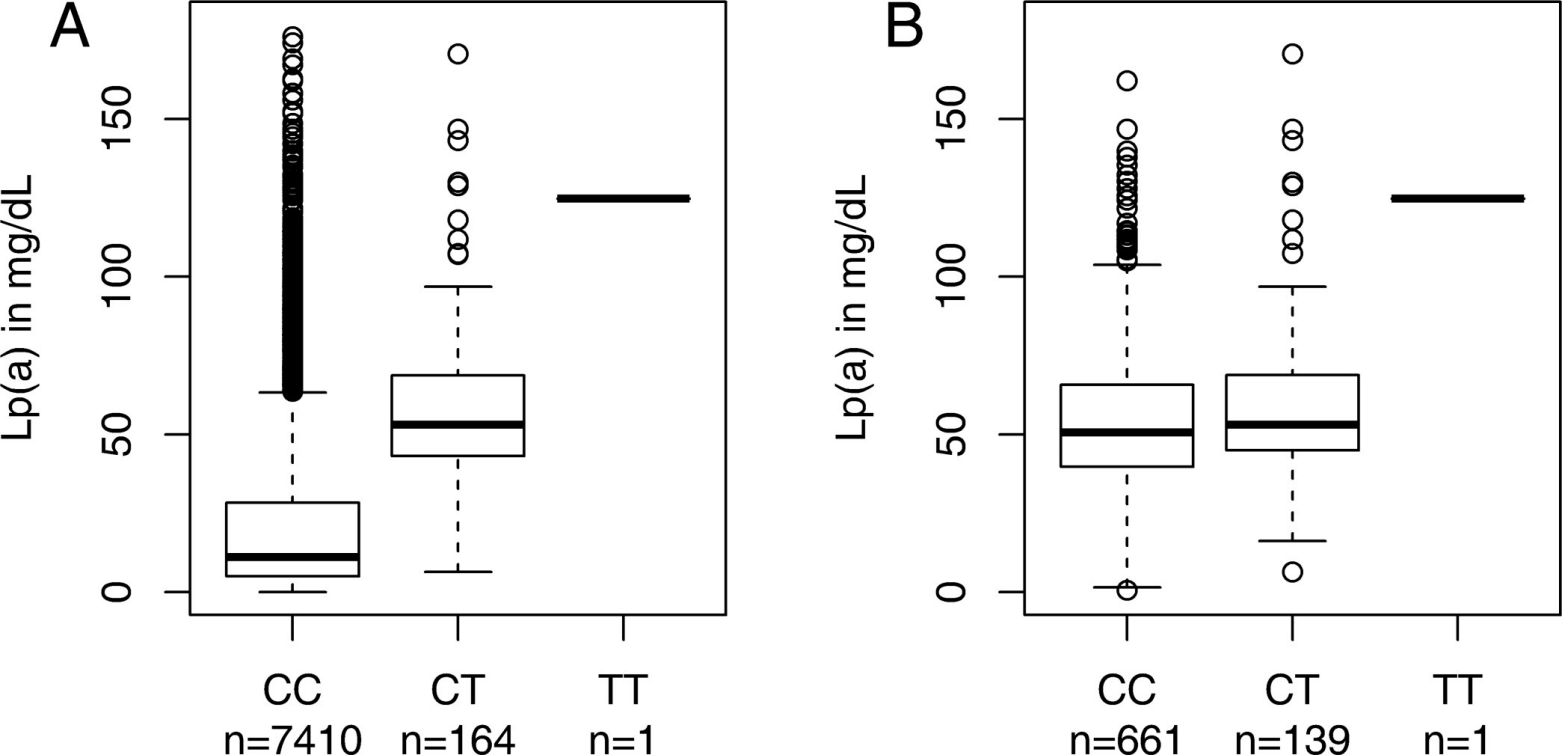
Distribution of Lp(a) stratified for genotypes of SNP rs76735376. In all participants of all three cohorts (panel A) and in subgroup of participants with at least one minor allele of rs10455872 (panel B).

The minor allele of rs76735376 was associated with increased Lp(a) values (Fig 2) with consistent effect sizes in all three cohorts (p = 1.01e-59, Table 2). Each copy of the minor allele was associated with an increase of Lp(a) concentrations by 37 mg/dL. The SNP explained 3.45% of the variance of Lp(a) concentrations. One part of the SNP effect seems to be due to correlation with apo(a) isoforms. As already observed for the methylation levels, isoforms 18–20 are overrepresented in carriers of the minor allele (T) (S6A Fig). Additional adjusting for apo(a) isoforms leads to a reduction of the effect size (β = 20.72; p = 1.72e-21, Table 2).

**Table 2:** Association analysis of rs76735376 on Lp(a) concentrations.

Study	Results of linear regression				
	n	beta	se	p-value	Variance explained
**Adjusted for age and sex**					
KORA F4	2986	36.42	2.81	3.23e-25	0.0384
KORA F3	3080	38.06	3.14	2.89e-27	0.0342
SAPHIR	1446	36.84	5.23	9.81e-11	0.0273
Combined*	**7512**	**37.12**	**1.96**	**1.01e-59**	**0.0346**^†^
**Adjusted for age, sex and apo(a) isoforms:**					
KORA F4	2986	19.65	2.49	1.37e-10	0.0062
KORA F3	3080	22.23	2.77	4.70e-09	0.0109
SAPHIR	1446	20.11	4.62	8.09e-05	0.0104
Combined*	**7512**	**20.71**	**1.73**	**1.73e-21**	**0.0089**^†^
**Adjusted for age, sex and rs10455872:**					
KORA F4	2857	5.94	2.82	0.1257	0.0026
KORA F3	2998	14.60	3.15	2.50e-04	0.0030
SAPHIR	1426	2.74	5.13	0.5740	0
Combined*	**7281**	**8.95**	**1.96**	**4.00e-04**	**0.0016**^†^
**Adjusted for age, sex, rs10455872 and apo(a) isoforms:**					
KORA F4	1831	2.94	2.59	0.2588	**0**
KORA F3	1976	9.66	2.83	0.0156	**0.0017**
SAPHIR	1413	1.12	4.71	0.8120	**0**
Combined*	**7220**	**5.38**	**1.80**	**0.0463**	**0**^†^

Models are adjusted for age and sex and additionally also for apo(a) isofroms and/or rs10455872. The p-value and variance explained are derived from the log-transformed model, whereas beta estimate and standard error (se) are derived from the model on the original scale of Lp(a) to ease interpretability and refer to the minor allele (T) of rs76735376.

*Linear mixed effects model with “study” as random effect;

^†^weighted average of study-specific variance explained

### Association with the pentanucleotide-repeat (PNR) polymorphism

Since rs76735376 is in close vicinity to the well-known *LPA* pentanucleotide-repeat (PNR) polymorphism[30,38,39,53], we evaluated in KORA F4 and SAPHIR, whether the association of the PNR with Lp(a) concentrations can be explained by rs76735376 or vice versa. In both studies, 89% of all participants present one allele with 8 repeats of this PNR, whereas all carriers of the minor allele of rs76735376 have this repeat number at least once (S5 Table, p = 0.01, Fisher’s exact test in both studies combined). However, in a mixed effects model using data from both studies, the effect of the PNR on log-lp(a) (beta = -0.15, se = 0.04, p = 0.0003) is mediated only marginally by rs76735376 (results of PNR adjusted for rs76735376: beta = -0.14, se = 0.04, p = 0.0012). The SNP-effect is not affected at all by adding the PNR to the model (effect of SNP only in both studies on log-lp(a): beta = 1.55, se = 0.12, p = 3.82e-37; Adjusted for PNR: beta = 1.53, se = 0.12, p = 1.62e-36).

### Mediation analysis

Since rs76735376 was identified actually by chance in a genome-wide methylation analysis, we investigated whether the effect of the SNP on Lp(a) is mediated through the methylation signal. A formal mediation analysis revealed no significant causal mediation effect of the methylation level of cg17028067 on log-Lp(a) (p = 0.13, S7 Fig). The total effect of the SNP on Lp(a) is only marginally altered by inclusion of the beta-value of cg17028067 in the model. In a regression model with both, rs76735376 and cg17028067, only the SNP remains significant. This might indicate that the true causal factor is the base change at rs76735376 rather than the methylation level at cg17028067.

### Effect of rs76735376 on *LPA* transcription

*LPA* is expressed exclusively in liver tissue, which is hard to obtain for a large number of samples, especially if they need to be controlled for defined rare *LPA* genotypes. To assess whether rs76735376 indeed affects *LPA* expression in vivo, we performed a lookup in the data of the GTEx consortium V8 data [54]. GTEx is a multinational project providing both genome-wide SNP data and complete transcriptome data for >17,000 individuals and 54 different tissues, including eQTL data from 208 liver samples. The minor allele rs76735376 was significantly associated with increased *LPA* expression in liver tissue (beta(se) = +0.64 (0.18), p = 0.00038) despite only 15 heterozygous carriers in the data. Accordingly, bioinformatic transcription factor binding analysis using TRANSFAC[55] revealed a direct DNA transcription factor binding site for Oct factors *POU2F1* and *POU5F1* (also known as Oct1 and Oct3/4) within the T -allele of the rs76735376 SNP but not the C- or 5-mC allele (S3 Fig). Moreover, 5 bases besides the Oct binding site, a *CEBPB* was predicted (S3 Fig). Pilot experiments using electrophoretic mobility shift assays (EMSA) with probes encompassing the SNP site with C, 5-mC and T allele oligonucleotides suggests that *CEBPB* but not the Oct factors *POU2F1* and *POU5F1* indeed bind to both the C and 5-mC allele, but not to the T-allele (S8 Fig).

### Integrating GWAS results with eQTL results and further association analyses

eQTL association results were further derived for all SNPs in LD (r^2^≥0.1) with rs76735376 (10 SNPs). S9 Fig shows a scatterplot of these SNPs between p-values derived from a genetic association analysis on Lp(a) versus eQTL p-values. The maximum (-log10)-transformed p-values for both analyses can be found the SNPs rs10455872[6] and rs118039278[23]. Since both are in perfect LD (r^2^ = 1), we included only rs10455872into the further analysis. While r^2^ between rs76735376 and rs10455872 is only 0.1 and 0.17 in KORA F3 and KORA F4 due to different allele frequencies, almost all carriers of the minor allele of rs76735376 carry also at least one minor allele of rs10455872 (S6 Table), leading to a D’ of 0.71 in KORA F3 and 0.95 in KORA F4. To evaluate, whether the association signal of rs76735376 can be explained by rs10455872, the linear regression model on Lp(a) was further adjusted for rs10455872, which reduced the effect size to ≈+9 mg/dL (p = 4e-04, Table 2).

Since rs76735376 coincides with isoforms 18 to 21 and since also rs10455872 was shown to be correlated with small isoforms (S6B Table), the analysis was further adjusted for both rs10455872 and apo(a) isoforms, leading to an increase of Lp(a) by 5.38 mg/dL (p = 0.0463) for each minor allele of rs76735376 (Table 2).

## Discussion

Lp(a) is a major genetically determined cardiovascular risk factor. Research on Lp(a) has experienced two revivals: the first by genetic studies providing strong support for a causal association with cardiovascular outcomes and the second, more recently, due to Lp(a)-lowering agents becoming available in the form of PCSK9 inhibitors and mRNA antisense therapy. Especially the advent of an antisense oligonucleotide therapy capable of reducing Lp(a) by >80%[56] now puts the validation of the “Lp(a) hypothesis” within reach[57]. Different studies reported that Lp(a) would have to be reduced by 50 mg/dL to even above 100 mg/dL [58–61] to produce the same risk reduction as LDL-C reduction by 1 mmol (38.67 mg/dL).

Despite the strong impact of Lp(a) on CVD risk, many controversies and uncertainties remain about its metabolism and regulation[62]. The association of Lp(a) concentrations with the apo(a) isoform size is well accepted, but far from being simple and linear[1,63]. Even within the same isoform group, levels may vary by 200-fold[22], suggesting the existence of modifier variants that act on top of the apo(a) isoforms. Some of these have been identified[24,25,28], but they all act via disturbing protein synthesis.

Regulatory variants and elements are less defined. Therefore, we performed the first genome-wide DNA methylation study for Lp(a). This identified the SNP rs76735376 located in a CpG site in the *LPA* promoter. Each minor allele abolishes a methylation site in a CpG dinucleotide and is associated with an increase in Lp(a) of about 37 mg/dL and 9 mg/dL when restricted to individuals with 18–20 KIV repeats who are the main carriers of this methylation site. To dissect the effect of the base from the methylation site we performed a statistical mediation analysis which indicated that the effect observed in the association study is driven by the base change and not by the methylation status of the locus. This was supported by pilot EMSA experiments using the three possible allele states C, 5m-C and T), which showed no clearly defined difference between the C and the 5m-C allele, but a difference between the C and T alleles.

The location in the promoter suggests an effect on the transcriptional regulation of *LPA*. While Lp(a) is exclusively expressed in the liver and native tissue is hard to obtain in numbers sufficient to account for the low MAF of rs76735376. The recent large-scale multi-tissue and eQTL profiling initiative GTEx, however, provides a convenient way to still investigate the effects of rs76735376 on *LPA* expression. Indeed, a look-up for in the GTEx data revealed that the rs76735376 T-allele is associated with significantly increased *LPA* expression, which was in line with the observed increase of Lp(a) concentrations in these individuals.

Interestingly, rs76735376 was located close to the *LPA* pentanucleotide repeat (PNR). This led us to speculate that it might be the causal factor underlying the previously reported[30], but not clearly replicated[31] impact of the PNR on *LPA* expression. This was, however, not the case as the association signal at rs76735376 was only marginally affected by including the PNR in the model and vice versa. Of note, all carriers of the minor allele of rs76735376 carried a PNR allele with 8 copies and virtually all minor allele carriers presented an apo(a) allele with 19 to 20 KIV repeat (S6 Fig). Rs76735376 therefore adds to the other putative functional SNPs in *LPA*, which are confined to certain isoform ranges[28,36,64].

This is also true for the widely studied SNP rs10455872 [6], which coincides with rather small isoforms, too. Integrating eQTL association results with genetic association results, pinpointed to rs10455872 as the potential trigger of the effect of rs76735376. Indeed, adjusting for both, rs10455872 and the apo(a) isoforms diminished the effect size of rs76735376 from 37.1 mg/dL to 5.38 mg/dL. Although the p-value for this analysis is still <0.05, it cannot be deemed significant in a strict sense. The methylation signal was identified via a genome-wide methylation analysis and the samples for the subsequent genotype association analysis were only partly independent and therefore, all results have to be accounted for this multiple testing situation.

Altogether, our results indicate that a major part of the effect of rs76735376 can be explained by correlation with rs10455872 and apo(a) isoforms. This underscores the complex genetic make-up of the Lp(a) trait, consisting of an intricate interplay between the KIV-2 repeat number, transcriptional regulation by enhancer and promoter regions, modifier SNPs, which may be in turn confined only to certain isoform sizes, and trans-regulating factors like apoE[23,65]. Given the large impact of the underlying apo(a) isoform on Lp(a) levels, a full assessment of variants influencing Lp(a) is likely to require complete knowledge about the isoforms of the investigated samples. Failure to account for the isoforms has been shown to even potentially mask true genetic effects[28,36].

In light of these observations are our findings still relevant and worth being discussed? Indeed, our study highlights several important messages for the community. First, effect size from the isoform- and rs10455872-adjusted analysis is still 5.69 mg/dL, which is about 50% of the median level of Lp(a) and therefore cannot be deemed to be irrelevant. The only person homozygous for the rare allele has an Lp(a) level of 124.75 mg/dL, which corresponds to the 99.4% percentile. Therefore it cannot be excluded that this SNP still might have some independent function or following a recessive mode of inheritance.

Secondly, rs10455872 explains a considerable part of the effect of rs76735376, despite presenting an r^2^ of only 0.1 and 0.17. However, the high D’ values of 0.71 and 0.95 are sufficient to take up most of the signal. Indeed the D’ values are often neglected when evaluating LD structures and it is often implicitly assumed that only a high r^2^ can trigger an indirect signal. We exemplify here that this is not always the case. Moreover, it is also important to note how promising functional data may not necessarily translate to a true impact on the phenotype. Indeed mRNA levels do not necessarily always correlate strongly with the protein levels[66].

The third message is a rather technical one. Indeed, when we originally set out to investigate the impact of DNA methylation on Lp(a), the quality control procedure was supposed to purge any CpG sites affected by a SNP by using imputed 1000Genomes genotype data filtered at 5% MAF to focus only on differential methylation but not common ASM-SNPs. However, rs76735376 escaped filtering because it was badly imputed and thus incorrectly reported with a MAF of 0.1%. Since such a low MAF could not have triggered the association signal observed, we genotyped it de-novo, revealing a ten times higher true MAF. This MAF is now confirmed also by new reference datasets that became available only very recently (like TOPMed[67], https://bravo.sph.umich.edu/freeze5/hg38/). This exemplifies that, when drilling down from GWAS data to single SNPs, comparing the MAF from still widely used 1000 Genomes imputed data with that in the most recent resources like TOPMed can help to detect inconsistencies, which could otherwise bias the results if not handled properly.

Our study presents some limitations which need to be mentioned. Firstly, the methylation dataset was derived from peripheral blood, while *LPA* expression is limited to hepatic tissue. This is an unavoidable limitation of population-scale genome wide methylation analyses since hepatic tissue is not accessible at the sample numbers required for genome-wide studies. Indeed, even the GTEx initiative, which contains data from ≈17,000 individuals and 54 different tissues (GTEx V8 as of December 2019), reports only 228 liver samples, thereof only 208 having also SNP genotype data available. While methylation is clearly a tissue-specific marker, studies also have shown that a consistent portion of the DNA methylation patterns is still conserved across tissues[68,69]. Therefore, while we might have missed some very tissue-specific regulators, we can be confident that a consistent portion of methylation-based regulation has been assayed. Importantly, the methylation status has been validated independently by Sanger sequencing. Unfortunately, due to limited amounts of biobank material available for bisulfite conversion, we acknowledge that we could perform this technical validation could only in a relatively low number of samples (n = 8).

Secondly, our dataset was of Caucasian origin. Given the known differences in allele frequencies and effects of *LPA* SNPs between ethnicities, investigations in other populations are required to establish the meaning of our findings in other ethnicities[70,71].

Thirdly, it needs to be mentioned that also GTEx presents limitations[72]. Most relevant for this work is that even eQTL signals still represent a form of association. Therefore, ultimately only functional studies like CRISPR editing can provide assess the true causality of SNPs on expression. They are, however, currently limited by the fact that *LPA* expression is blunted in cell culture for still unknown reasons and that the relationship of mRNA expression to secerned Lp(a) is not fully elucidated[73].

In conclusion, this study represents a first step assessing the epigenetic regulation of Lp(a). We showed that the analyzed region of the Lp(a) promoter is subjected to DNA methylation and that the identified CpG site cg17028067, respectively rs76735376, is associated with increased Lp(a) concentrations. This newly identified variant, however, is another example of the complex LD structures in the Lp(a) gene, since, despite clear eQTL signals, major parts of the effect could be finally explained by correlation with a complex interplay of apo(a) isoforms and rs10455872 genotype. This exemplifies the intricate genetic architecture of Lp(a).

## Materials and methods

### Study design and populations

The overall study design is outlined in S1 Fig. Three population-based studies were available for this analysis: The KORA F3, KORA F4 and SAPHIR studies. The KORA study (Cooperative health research in the region of Augsburg) consists of independent, non-overlapping, population-based samples from the general population living in the region of Augsburg, Southern Germany, and was conducted in the years 2004/2005 (KORA F3) and 2006 to 2008 (KORA F4)[74]. A total of 3080 subjects with ages ranging from 32 to 81 years participated in the KORA F4 examination (stratified by sex-and age-groups). The genome-wide DNA methylation patterns were determined in a random subgroup of 1802 subjects (n = 1724 in analysis dataset with non-missing values in all relevant variables). In KORA F3 (n = 3184, age-range between 35 and 84 years), DNA methylation data were available from 484 subjects (comprised of 250 smokers and 250 non-smokers; 16 samples are missing due to data preprocessing or missing values in one of the relevant variables). For additional de novo genotyping, samples and data from 3080 KORA F3 and 2986 KORA F4 study participants were available, as well as samples from the SAPHIR study. For the SAPHIR study (Salzburg Atherosclerosis Prevention Program in subjects at High Individual Risk), participants were recruited by health screening programs in large companies in and around the city of Salzburg, Austria (n = 1770, age-range 39–67 years). Genotypes and relevant phenotype data were available for 1446 participants. All study participants provided a signed informed consent and the studies were approved by their respective local ethics committee (Bayerische Landesärztekammer for the KORA studies, Ethical Committee of Salzburg for SAPHIR). The PNR was measured in a subset of the KORA F4 (n = 2956) and SAPHIR (n = 1428) studies.

### Genome-wide DNA methylation

Genome-wide DNA methylation patterns in the discovery and replication cohorts were analyzed using the Infinium HumanMethylation450 BeadChip Array (Illumina) and DNA derived from peripheral blood. KORA F3 and F4 samples were measured and processed separately [75]. KORA F3/F4 samples were processed on 20 respectively 7 96-well plates in 9 respectively 4 batches; plate and batch effects were investigated using principal component analysis and eigenR2 analysis[76]. DNA methylation data were preprocessed following the CPACOR pipeline[77]. Background correction was performed using the R package minfi, v1.6.0[78]. Signals with detection p-values (the probability of a signal being detected above the background) ≥ 0.01 were removed, as they indicate unreliable signals, as were signals summarized from fewer than three functional beads on the chip. Observations with less than 95% of CpG sites providing reliable signals were excluded.

To reduce the non-biological variability between observations, data were normalized using quantile normalization (QN) on the raw signal intensities: QN was performed on a stratification of the probe categories into 6 types, based on probe type and color channel, using the R package limma, v3.16.5[79].

The percentage of methylation at a given cytosine is reported as a beta-value, which is a continuous variable between 0 and 1 corresponding to the ratio of the methylated signal over the sum of the methylated and unmethylated signals of the particular cytosine site. Since it was the primary idea to detect the effect of differential methylation on Lp(a) and since probe binding might be affected by SNPs in the binding area, sites representing or being located in a 50 bp proximity to SNPs with a minor allele frequency (MAF) of at least 5% were excluded from the data set[80]. Nine CpG sites in the *LPA* gene locus were present on the microarray (S1 Table).

### SNP validation and *LPA* pentanucleotide repeat genotyping

KORA F4 samples were genotyped using the Affymetrix Axiom chip array. Genotypes were called with the Affymetrix software and were annotated to NCBI build 37. Imputation was performed with IMPUTE v2.3.0 using the 1000G phase1 (v3) reference panel[81]. Additional de novo genotyping for rs76735376 was performed in KORA F3, KORA F4 and the SAPHIR using a commercial Taqman assay (ThermoFisher Scientific).

Due to the low MAF, samples being heterozygous and homozygous for the rare allele were confirmed by Sanger sequencing using the primers 5’-TACAGGACAGAGACTAACT-3’ and 5’-GCATAGTATCAATCTTTCCG-3’. The PCR conditions were: Qiagen Taq DNA Polymerase (#201207), 0.2 mM dNTP, 0.5 μM primer, 1x Qiagen Q-Solution reagent, 40 ng input DNA amount. Cycling conditions were 94°C 3’ initial denaturation, 40 cycles of 94°C 30 sec, 60°C 30 sec, 72°C 30 sec, 72°C 10 minutes final elongation. Sequencing was done using ABI BigDye 1.1 chemistry (Thermo Fisher Scientific Scientific, Waltham, MA, USA).

The *LPA* pentanucleotide repeat (PNR) is a microsatellite with 5-12 repeats of a TTTTA element located at hg19:chr6:161,086,617–161,086,663 (according to the RepeatMasker tool in the UCSC Genome Browser[82]) and thus only ≈50 bp downstream of rs76735376. It has been genotyped by fragment analysis in SAPHIR and KORA F4. Details of the PNR genotyping via fragment analysis are given in reference[53]. In brief, a 181 bp PCR fragment encompassing the *LPA* pentanucleotide repeat was amplified in KORA F4 (data available in n = 2956) and SAPHIR (data available in n = 1429) in 384 well plates in a total volume of 5 μl with 2.5 μl Qiagen Multiplex PCR Plus Kit (Qiagen, Hilden, Germany) and 1 μl Qiagen Q-solution using a Yakima Yellow-labelled primer. Fragment length was detected by fluorescent capillary fragment analysis on an ABI 3730s Genetic Analyzer and all data analyzed using the GeneMapper software (all Thermo Fisher Scientific). Data was quality controlled for null allele overrepresentation and deviation from Hardy-Weinberg Equilibrium using Micro-Checker[83] (http://www.microchecker.hull.ac.uk/) and ARLEQUIN (http://cmpg.unibe.ch/software/arlequin3/) [84].

### Bisulfite sequencing

Bisulfite Sanger sequencing was used to validate the methylation signal at rs76735376 and map the methylation status of other CpG sites nearby in 8 selected samples from SAPHIR (4 homozygotes for the major allele and 4 heterozygotes). S2 Fig provides an overview of the region with location of the PNR, the primers, the amplicons and the CpG affected by rs76735376, as well as the neighboring CpG sites covered (respectively not covered) by our bisulfite sequencing.

500 ng sample DNA were bisulfite treated using the kit EZ DNA Methylation-Lightning Kit (Zymo Research, Irvine, CA, USA) and eluted in two times 10 μl. 1 μl eluted converted DNA was used as template for PCR. The region was amplified using the DNA Polymerase of the Agena PCR Accessory and Enzyme Set (Agena Bioscience, San Diego, CA, USA). PCR conditions were: 0.4 u Agena DNA Polymerase, 0.5 mM dNTP, 0.2 μM primer, 2 mM MgCl_2_, 1 μl converted DNA. Cycling conditions were 94°C 4 minutes initial denaturation, 45 cycles of 94°C 20 sec, Annealing 30 sec, 72°C 60 sec, final elongation at 72°C 3 minutes. Primers for bisulfite-converted DNA were designed using the Agena EpiTyper Software Suite (see S2 Table for sequences and annealing temperature). All sequencing was done using ABI BigDye 1.1 chemistry.

### Measurement of Lp(a) concentrations and apo(a) isoform size

Lp(a) concentration were determined in plasma using the same standardized sandwich ELISA assay in all samples. The assay has been described in detail previously[63,85]. In brief, ELISA plates were coated with an affinity-purified rabbit anti-human apo(a) antibody in a final concentration of 5 μg/ml in 1x PBS containing 1 mg/mL NaN_3_. The plates were incubated with 100 μl antibody dilution (3 h, 37°C), washed three times (1x PBS + 0.05% v/v Tween-20) and blocked with 200 μl 0.1*%* w/v casein in 1x PBS pH 7.3 (30 min, 37°C). To ensure measuring each sample within the linear range of optical density, all samples were diluted into the ELISA plate twice (1:150 and 1:1,500 in Assay Buffer (Microcoat, Bernried, DE) out of 1:30 and 1:1000 predilutions in 1x PBS, pH 7.3). A 7-point standard curve ranging from 0.32 mg/dL to 5 μg/dL was created (with an additional blank representing the zero point). Duplicate determinations of four reference samples were used as longitudinal inter-assay controls. The coated plates were incubated with the analyte for 1 hour at 37°C. After several washing steps, the bound Lp(a) was detected using a horseradish-peroxidase-conjugated monoclonal antibody (1A2; in 0.1% w/v casein, 1x PBS, pH 7.3) directed against the KIV-2 domain and not cross-reacting with plasminogen[86] and the Blue Star TMB substrate (Adaltis, Guidonia Montecelio, IT) (30 minutes incubation at room temperature). Reaction was stopped by adding 50 μl 0.5 M sulfuric acid. Measurement of the absorption (dual wavelength, analyte: 450 nm, reference: 690 nm) was done using a Microplate Reader (BioRad Benchmark Plus, Bio-Rad Laboratories, Hercules, USA) and concentrations were calculated based on the standard curve (expressed as mg/dL). All dilution and pipetting steps were done using liquid handling robotics (TECAN, Männedorf, CH).

Lp(a) isoform was determined by Western blotting in all samples as described[63]. In brief, 150 ng Lp(a) as determined in the ELISA were loaded on a 1.46*%* agarose gel with 0.08*%* SDS and separated for 18 h at 0.04 A (constant current). A size standard consisting of a mixture of five plasma samples collected from individuals expressing each only one apo(a) isoform (13, 19, 23, 27, 35 KIV repeats determined by Pulsed Field Gel Electrophoresis) was applied in every seventh well of the gel. The gel was subsequently blotted to a PVDF membrane (Immobilion-P, Millipore, Darmstadt, DE) by semi-dry blotting (Perfect Blue Semi Dry Blotter, VWR, Vienna, A). After blocking (85 mM NaCl, 10 mM TRIS, 0.2*%* Triton X-100, 1*%* BSA; ≥30 min at 37°C), the membrane was incubated with horseradish-peroxidase-conjugated 1A2 antibody (1A2 concentration was 168 ng/ml in Buffer C) for 2 h at room temperature on a shaker. After washing with TTBS (3x 15 min), ECL substrate (WesternBright Chemilumineszenz Spray, Biozym, Hessisch Oldendorf, DE) was added and signals recorded on an Amersham Hyperfilm^™^ ECL^™^ (GE Healthcare, Vienna, A).

In heterozygous individuals, the smaller (and thus the commonly Lp(a) concentration-determining) of the two alleles was used for isoform-adjustment of statistical models.

### Statistical methods

#### Genome wide methylation analysis

Linear regression models were applied to evaluate the association between DNA methylation beta values and Lp(a). Lp(a) levels were log-transformed to account for the skewed distribution. Age, sex and the first 20 principal components of the Illumina control probes (to adjust for technical confounding) were included as potential confounders. In addition, since whole blood DNA samples were used, cell heterogeneity had to be considered as a confounder. As no measured cell count information was available, sample-specific estimates of the proportion of the major white blood cell types were obtained using a statistical method described by Houseman et al[87]. To correct for multiple comparisons, a Bonferroni corrected significance level of 1.03e-07 (= 0.05/485512) was used. Genome-wide significant findings were replicated in KORA F3 using the same adjustment model as in the discovery stage. Fixed-effects inverse-variance meta-analysis was applied to summarize the findings in KORA F3 and F4. The analyses were repeated additionally adjusting for apo(a) isoforms or in subgroups of apo(a) isoforms. Since it was shown previously that differential methylation can be a consequence of variation of lipid levels [88], the analyses were also adjusted for LDL cholesterol (LDL-C).

#### SNP association and mediation analysis

Association of the identified SNP with log-transformed Lp(a) was evaluated in all three studies individually (KORA F3, KORA F4, SAPHIR) using a linear regression model and in all three studies combined using a linear mixed effects model with “study” as a random effect and adjusting for age and sex. The genotypes of the SNP were additively coded, that is, effect sizes refer to one copy of the minor allele. This analysis was repeated additionally adjusting for apo(a) isoforms or in subgroups of apo(a) isoforms. Furthermore, as a consequence of the results of the analysis described in the next paragraph (“Integrating GWAS results with eQTL results”), the association analysis was further adjusted for rs10455872 and in subgroups of individuals with at least one minor allele of rs10455872.

In addition to the models with log-transformed Lp(a) values as the dependent variable, which were used to ensure the normal-distribution assumption to derive the test-statistic and p-values, Lp(a) on its original scale was used. From these models, beta estimates and standard errors (se) were derived to ease interpretability of the effect sizes.

Due to the close vicinity of the cg-site/SNP with the well-known PNR polymorphism, the association of the PNR with Lp(a) concentrations was evaluated and whether this effect can be explained by the SNP or vice versa.

Since both, the identified SNP and the methylation site are significantly associated with Lp(a) and since the methylation level is a consequence of the allelic variation of the SNP, it might be conceivable that there is no direct effect of the SNP on Lp(a), but that this effect is mediated by the methylation levels. To assess whether the effect of rs76735376 on Lp(a) is mediated through methylation, a formal mediation analysis was applied using the R library “mediation”.

#### Integrating GWAS results with eQTL results

To evaluate, whether the association signal at rs76735376 may actually be derived from a stronger eQTL signal in LD with this SNP, a list of SNPs with r^2^≥0.1 with rs76735376 was derived. This SNP-list was integrated with all SNPs, for which eQTL data from GTEx (see next paragraph) are available and with results derived from a genome-wide association study (GWAS) on Lp(a). The GWAS results are based on the KORA F4 study (n = 2926) and were part of a published meta-analysis[23]. Since rs76735376 was not well imputed in this dataset, results for rs76735376 were replaced with the results from the de novo genotyped SNP as described before in “SNP validation and LPA pentanucleotide repeat genotyping”. All other results are based on 1000G imputed SNPs as described in [23].

### Follow-up in GTEx and transcription factor binding site prediction

The effect of rs76735376 on *LPA* gene expression in native liver tissue was assessed using the GTEx[89] data (www.gtexportal.com), which contains expression quantitative trait locus (eQTL) data with whole genome SNP data and whole transcriptome RNA-Seq data for 208 liver samples. The correlation of rs76735376 genotype with *LPA* expression in liver was extracted from the GTEx V8 dataset by downloading all pairwise SNP-expression correlations in liver tissue and using the SNP-ID “chr6_160665684” (chromosome and position of rs76735376 in hg38) and the Ensembl gene identifier for *LPA* “ENSG00000198670” to extract the data for rs76735376 and *LPA*. The dataset is available at https://console.cloud.google.com/storage/browser/_details/gtex-resources/GTEx_Analysis_v8_QTLs/GTEx_Analysis_v8_eQTL_all_associations/Liver.allpairs.txt.gz, accessible via the link “eQTL Tissue-Specific All SNP Gene Associations” at https://gtexportal.org/home/datasets, respectively at https://console.cloud.google.com/storage/browser/gtex-resources (accessed on November, 26^th^ 2019)

TRANSFAC (http://genexplain.com/transfac/) [55], a database predicting the genomic binding sites and DNA-binding profiles of eukaryotic transcription factors, was used in order to determine transcription factor binding sites at the genomic region of rs76735376.

### Gel mobility shift assay

To perform a pilot validation of the mediation analysis results, single-stranded oligonucleotides spanning the SNP site were synthesized by Eurofins MWG Operon and then annealed to generate double-stranded probes. The single-stranded oligonucleotide sequences for the C, T and 5-mC allele of rs76735376 respectively are shown in S3 Fig. 5-mC bases were introduced directly by the oligonucleotide provider during oligonucleotide synthesis. Annealing was performed by heating a mix of both oligos (2.5 μg in 100 μl with 1X annealing buffer: 20 mM TRIS ph 8.0, 1 mM EDTA, 50 mM NaCl) to 65° C for 5 minutes and slowly cooling down in ice water. Binding reactions for super shift assays were performed for 30 min at 4–8°C using the Binding Buffer B-1 (Active Motif 37480) together with Stabilizing Solution D (Active Motif 37488) containing either 5 μg human liver or embryonic kidney nuclear extracts (Active Motif 36042; 36033), 2 μg of either anti c/EBPbeta (*CEBPB*) (Santa Cruz Biotechnology; sc-150x), anti-Oct-1 (*POU2F1*) (Santa Cruz Biotechnology; sc232) plus anti-Oct-3/4 (*POU5F1*) (Santa Cruz Biotechnology; sc5279) or anti-Nfatc1 (*NFATC1*) as negative control (Santa Cruz Biotechnology; sc7294). Binding reactions with 3 × 10^5^ cpm of labeled probe was performed for 20 min at 4°-8°C using Binding Buffer C-1 (Active Motif 37484) together with Stabilizing Solution D (Active Motif 37488). Samples were separated on a 4% native polyacrylamide gel in 0.5 × TBE for 3 h at 250 V. For competition assays, 10-fold unlabeled cold oligonucleotides identical to the radioactively labeled non-methylated Oligo2 LPA_EMAS2 or the radioactively labeled methylated Oligo6 LPA_EMAS2 probes were added to the binding reaction in addition.

## Supporting information

S1 Table CpG sites in the LPA gene locus assayed by the illumina infinium HumanMethylation450 BeadChip array with position, location and the p-value of the first stage epigenome-wide analysis on log(Lp(a)). The location is given relative to the UCSC Genome browser hg38 annotation with a leading non-coding exon 1. See the introduction section of the main manuscript for an explanation of the issues with the LPA reference transcript annotation.
(PDF)

S2 Table Primer sequences. For Bisulfite Sanger sequencing primer the lower case letters denote tails that have been added by the design software to increase the annealing temperature of the primer. The PCR protocols are given in the Methods section of the manuscript. See S2 Fig for an overview of the target sequence.
(PDF)

S3 Table Study characteristics (Mean ±sd [25%,50%,75% Percentile] for quantitative variables, n(%) for gender. (PDF)

S4 Table Genotype frequencies of the de novo genotyped SNP rs76735376 in three cohorts. (PDF)

S5 Table Allele frequencies of the PNR in SAPHIR and KORA F4, separated for genotypes of rs76735376. (PDF)

S6 Table Frequencies of the combined genotype distributions of rs76735376 with rs10455872 in all three cohorts together. (PDF)

S1 Fig Flow chart of the study design. (PDF)

S2 Fig Overview of the region around rs76735376 with location of the LPA pentanucleotide repeat, the location of the amplicons, the location of the CpG affected by rs76735376 and neighboring CpG sites. Upper case: sequence regions, which could be covered with amplicons for bisulfite sequencing. Lower case: regions which could not be covered by amplicons for bisulfite sequencing. Blue background (light and dark): primer binding sites for bisulfite sequencing. Note that the sequence given here is the unconverted sequence. The primer given in S2 Table bind on the converted sequence and the complement strand of the strand shown here. Yellow background: primer binding sites of the sequences used for SNP validation by sequencing. Bold character: LPA pentanucleotide repeat. Green: CpG affected by rs76735376 (enlarged cytosine base). Position of rs76735376 is underlined. Pink: CpG in the region, which were amenable to sequencing. Purple: CpG in the region, which were not amenable to sequencing (not represented in any amplicon). Sequence is the bisulfite-converted sequence given 5’ to 3’ on the minus strand of human genome GRCh37/hg19 (i.e. the same direction as LPA transcription direction).
(PDF)

S3 Fig Oligos used for EMSA experiments. The bold case base gives the location of rs76735376. Green background: POU2F1/POU5F1 binding site, Green font: mutated POU2F1/POU5F1 binding site, Blue CEBPB binding site. The reverse primers (-r) are given in reverse orientation, as they are annealed to the forward oligos (-f).
(PDF)

S4 Fig Scatterplot showing the lower of both apo(a) isoforms (x-axis) per person in KORA F3 (in red) and KORA F4 (in black) versus the beta-value of cg17028067 (y-axis). (PDF)

S5 Fig **Panel A:** Representative results of bisulfite sequencing in two homozygotes for the major allele and two heterozygotes in the SAPHIR study. The blue peak represents the unconverted C-allele, indicating that the major part is methylated in CC carriers, whereas only a minor part is methylated in CT carriers. **Panel B:** Boxplots of the methylation level (expressed as beta-value) of cg17028067, stratified for genotypes in the KORAF4 study (panel B).
(PDF)

S6 Fig Distribution of the smaller of both isoforms, stratified for carriers (CT or TT) and non-carriers (CC) of the SNP rs76735376 rs76735376 (panel A) and carriers and non-carriers of rs10455872 (panel B)
(PDF)

S7 Fig Possible paths and results of mediation analysis. (PDF)

S8 Fig Electrophoretic mobility shift assay for rs76735376. rs76735376 modifies binding of the transcription factor CEBPB. Left panel: Diagram of the double-stranded oligos used for EMSA analysis containing either cytosine (red, Oligo2 LPA_EMSA2-C), thymine (Oligo4 LPA_EMSA2-T) or methyl-cytosine (6 LPA_EMSA2-5mC) oligo. The transcription factor binding sites for CEBPB (blue) and POU2F1/POU5F1 (green) as predicted by Transfac analysis, 52 of the rs76735376 probes are shown. Position weight matrix of the TF is shown. The arrow indicates the orientation of the sequence matrix in the oligo. Right panel: EMSA analysis of human liver nuclear extract hybridized to Oligo2 LPA_EMSA2-C, Oligo4 LPA_EMSA2-T or Oligo6 LPA_EMSA2-5mC. Binding specificity to specific transcription factors was performed by using super-shift antibodies (s-shift AB) for the POU factors or CEBPB. An NFATC1 antibody and excess of cold unlabeled oligonucleotide (cold comp.) were used as negative control. One representative experiment out of three is shown. Note that the free probe has already left the gel because a long run time in order was required to distinguish the super-shift bands.
(PDF)

S9 Fig Scatterplot of p-values from a GWAS on Lp(a) versus p-values from eQTL analysis. The x-axis shows p-values from a GWAS on Lp(a) (using data from the KORA F4 study), the y-axis p-values from eQTL analysis (GTEx consortium V8). The index SNP (rs76735376) is marked as purple triangle. Only those SNPs are shown, which show a r^2^ of ≥ 0.1 with the index SNP (LD information derived from 1000 genomes CEU). Color coding shows magnitude of LD.
(PDF)
