# Supplementary material for: A genome-wide analysis of DNA methylation identifies a novel association signal for Lp(a) concentrations in the LPA promoter
Source: PLoS One. 2020 Apr 28;15(4):e0232073. doi: 10.1371/journal.pone.0232073 (PMC7188291; doi:10.1371/journal.pone.0232073)
Supplement: S1 Table — The location is given relative to the UCSC Genome browser hg38 annotation with a leading non-coding exon 1. See the introduction section of the main manuscript for an explanation of the issues with the LPA reference transcript annotation. (PDF) [file pone.0232073.s001.pdf]

S1 Table: CpG sites in the *LPA* gene locus assayed by the Illumina Infinium HumanMethylation450 BeadChip Array with position, location and the p-value of the first stage epigenome-wide analysis on log(Lp(a)). The location is given relative to the UCSC Genome browser hg38 annotation with a leading non-coding exon 1. See the introduction section of the main manuscript for an explanation of the issues with the *LPA* reference transcript annotation.

**cg-ID**	**Chr**	**Position**	**location**	**p-value**
cg10234069	6	160952713	Exon 40	0.1193
cg14663533	6	160955178	Intron 38-39	0.9046
**cg17028067**	**6**	**161086715**	**Intron 1-2**	**6.04e-11**
cg10836120	6	161087339	Exon 1	0.1954
cg11975608	6	161087439	5´upstream	0.8437
cg17189167	6	161087506	5´upstream	0.4256
cg16960593	6	161087573	5´upstream	0.4193
cg27656787	6	161088246	5´upstream	0.3816
cg07177174	6	161088755	5´upstream	0.0104
