# Supplementary material for: A genome-wide analysis of DNA methylation identifies a novel association signal for Lp(a) concentrations in the LPA promoter
Source: PLoS One. 2020 Apr 28;15(4):e0232073. doi: 10.1371/journal.pone.0232073 (PMC7188291; doi:10.1371/journal.pone.0232073)
Supplement: S2 Table — For Bisulfite Sanger sequencing primer the lower case letters denote tails that have been added by the design software to increase the annealing temperature of the primer. The PCR protocols are given in the Methods section of the manuscript. See S2 Fig for an overview of the target sequence. (PDF) [file pone.0232073.s002.pdf]

S2 Table: Primer sequences. For Bisulfite Sanger sequencing primer the lower case letters denote tails that have been added by the design software to increase the annealing temperature of the primer. The PCR protocols are given in the Methods section of the manuscript. See Supplementary Figure 2 for an overview of the target sequence.

**Primer-ID**	**Sequence (5' – 3')**	**use**	**annealing temperature [°C]**
LPA_as_785_fw	aggaagagagTAGGAGGTGGAAGTTGTAGTGAGTT	bisulfite sequencing amplicon 1 and sequencing primer	66
LPA_as_323_rv	cagtaatacgactcactatagggagaaggctAAACCACTCACCCTCCTAAAATATC	bisulfite sequencing amplicon 1	66
LPA_sense_1211_fw	aggaagagagTGGGATGATTGGTATGTGTTTTTAT	bisulfite sequencing amplicon 2 and sequencing primer	63
LPA_sense_1694_rv	cagtaatacgactcactatagggagaaggctCCAAACCCTCTACCAAATACTACAC	bisulfite sequencing amplicon 2	63
LPA_fw_rs76735376	TACAGGACAGAGACTAACT	amplicon for validation of rs76735376 by sequencing	60
LPA_rw_rs76735376	GCATAGTATCAATCTTTCCG	amplicon for validation of rs76735376 by sequencing	60
LPA_sense_1377_fw	TTGGAAGGATTGATATTTTATAATATAATTTAT	sequencing primer for bisulfite DNA for amplicon 2	55
LPA_as_408_rv	CAAAAACTAACTACACACAATATCTAAAACT	sequencing primer for bisulfite DNA for amplicon 1	55
