# Supplementary material for: A genome-wide analysis of DNA methylation identifies a novel association signal for Lp(a) concentrations in the LPA promoter
Source: PLoS One. 2020 Apr 28;15(4):e0232073. doi: 10.1371/journal.pone.0232073 (PMC7188291; doi:10.1371/journal.pone.0232073)
Supplement: S3 Table — (PDF) [file pone.0232073.s003.pdf]

**S3 Table:**: Study characteristics (Mean ±sd [25%,50%,75% Percentile] for quantitative variables, n(%) for gender

| **Study** | **KORA F4**<br>**n=2986***<br>**n=1724†** | **KORA F3**<br>**n=3080***<br>**n=484†** | **SAPHIR**<br>**n=1446*** |
|---|---|---|---|
| Age, yrs | 56.1±13.3 [44,56,67]<br>61.0±8.9 [54,61,68] | 57.4±12.9 [46.8,57,67]<br>53.2±9.6 [46,54,61] | 51.0±6.0 [46,52,55] |
| Gender (female) | 1545 (51.7%)<br>881 (51.1%) | 1583 (51.4%)<br>232 (47.9%) | 466 (32.2%) |
| Lp(a), mg/dL | 21.7±24.6 [5.2,11.7,30.3]<br>22.4±25.1 [5.5,12.1,31.3] | 22.1±26.2 [4.9,11.2,28.6]<br>20.1±23.1 [5.2,10.2,25.2] | 23.9±27.4 [5.3,11.6,35.6] |
| Apo(a) isoforms, KIV repeats‡ | 26.8±6.0 [23,26,31]<br>26.8±5.9 [23,26,31] | 27.2±6.0 [23,27,31]<br>27.1±5.8 [23,27,31] | 26.9±6.2 [22,26,31] |
| LDL-C in mg/dL | 135.9±33.8[112,134,157.5]<br>140±35.2[115,138,15] | 128.1±32.6[105,126.5,148]<br>131.3±33.1[108,129,152] | 142.4±35.4[118,141,165] |
| HDL-C in mg/dL | 56.8±15.2[46,55,66]<br>56.5±14.6[46,55,65] | 58.8±17.1[46.8,56,69]<br>58.3±17.9[46,56,68] | 59.1±15.5[48,57,68] |
| Total cholesterol in mg/dL | 216.3±38.7[189,215,239]<br>221.9±39.2[195,220,247] | 218.3±39.9[191,216,243]<br>221.1±38.1[195,218,244] | 226.5±38.8[199,224,251] |
| Triglycerides in mg/dL | 123.3±85.8[71,105,149]<br>133.6±95.6[77,110,158] | 164.7±125.9[88,135,200]<br>164.9±122.4[90,134,195] | 126±89.3[72,101,151] |

*First line in each cell: dataset available for SNP association analysis (with non-missing genotype, age, sex and Lp(a));
†Second line in each cell: subset available for methylation analysis (with no missing values in primary linear regression model);
‡ in heterozygous individuals the smaller of both isoforms per person is used
