# Supplementary material for: A genome-wide analysis of DNA methylation identifies a novel association signal for Lp(a) concentrations in the LPA promoter
Source: PLoS One. 2020 Apr 28;15(4):e0232073. doi: 10.1371/journal.pone.0232073 (PMC7188291; doi:10.1371/journal.pone.0232073)
Supplement: S4 Table — (PDF) [file pone.0232073.s004.pdf]

S4 Table: Genotype frequencies of the de novo genotyped SNP rs76735376 in three cohorts.

**Study**	**Number of genotypes and frequencies**			**Minor allele frequency**
	**CC**	**CT**	**TT**	
KORA F4	2912 (0.975)	74 (0.025)	0 (0)	0.012
KORA F3	3016 (0.979)	63 (0.020)	1 (0.0003)	0.011
SAPHIR	1419 (0.981)	27 (0.019)	0 (0)	0.009
