# Supplementary material for: A genome-wide analysis of DNA methylation identifies a novel association signal for Lp(a) concentrations in the LPA promoter
Source: PLoS One. 2020 Apr 28;15(4):e0232073. doi: 10.1371/journal.pone.0232073 (PMC7188291; doi:10.1371/journal.pone.0232073)
Supplement: S5 Table — (PDF) [file pone.0232073.s005.pdf]

S5 Table: Allele frequencies of the PNR in SAPHIR and KORA F4, separated for genotypes of rs76735376.

	SAPHIR		KORA F4	
**PNR allele [n repeats]**	**PNR allele Carriers, n (%) in CC-carriers**	**PNR allele Carriers, n (%) in CT-carriers**	**PNR allele Carriers, n (%) in CC-carriers**	**PNR allele Carriers, n (%) in CT-carriers**
4	1 (0.07)	0 (0)	0 (0)	0 (0)
5	0 (0.00)	0 (0)	5 (0.17)	0 (0)
6	7 (0.50)	0 (0)	4 (0.14)	0 (0)
7	14 (1.00)	0 (0)	19 (0.66)	0 (0)
8	1250 (89.16)	27 (100)	2570 (89.17)	74 (100)
9	103 (7.35)	0 (0)	202 (7.01)	0 (0)
10	27 (1.92)	0 (0)	82 (2.84)	0 (0)
