# Supplementary material for: A genome-wide analysis of DNA methylation identifies a novel association signal for Lp(a) concentrations in the LPA promoter
Source: PLoS One. 2020 Apr 28;15(4):e0232073. doi: 10.1371/journal.pone.0232073 (PMC7188291; doi:10.1371/journal.pone.0232073)
Supplement: S6 Table — (PDF) [file pone.0232073.s006.pdf]

S6 Table: Frequencies of the combined genotype distributions of rs76735376 with rs10455872 in all three cohorts together.

	rs10455872			∑
**rs76735376**	**AA**	**AG**	**GG**	
**CC**	6478	661	10	7149
**CT**	21	134	5	160
**TT**	0	1	0	1
∑	6499	796	15	
