# Supplementary figures and images for: A genome-wide analysis of DNA methylation identifies a novel association signal for Lp(a) concentrations in the LPA promoter
Source: PLoS One. 2020 Apr 28;15(4):e0232073. doi: 10.1371/journal.pone.0232073 (PMC7188291; doi:10.1371/journal.pone.0232073)

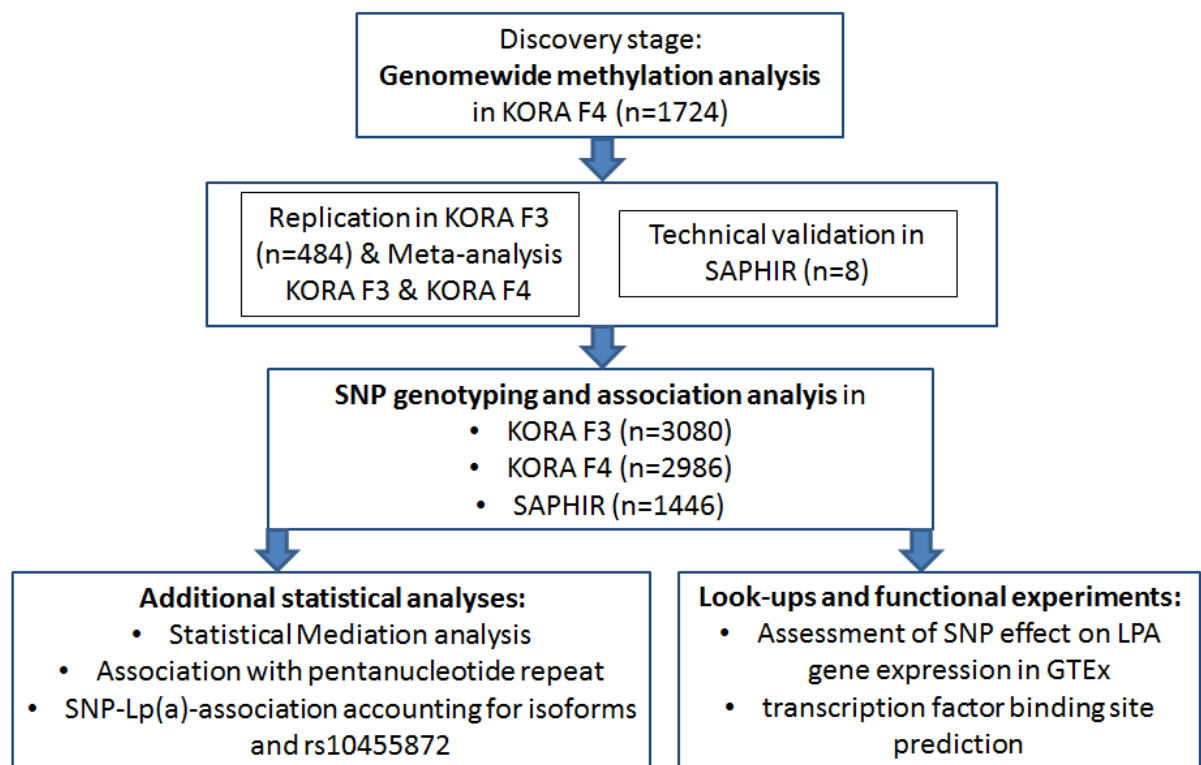

**S1 Fig: Flow chart of the study design**

Supplement: S1 Fig — (PDF) [file pone.0232073.s007.pdf]

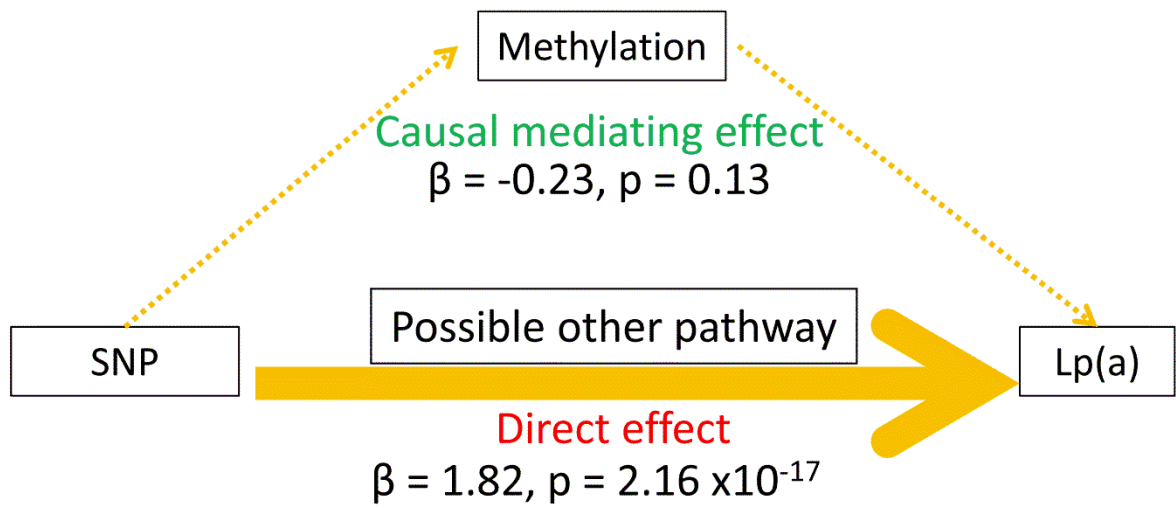

**S7 Fig:** Possible paths and results of mediation analysis.

Supplement: S7 Fig — (PDF) [file pone.0232073.s013.pdf]
