# Supplementary material for: A genome-wide analysis of DNA methylation identifies a novel association signal for Lp(a) concentrations in the LPA promoter
Source: PLoS One. 2020 Apr 28;15(4):e0232073. doi: 10.1371/journal.pone.0232073 (PMC7188291; doi:10.1371/journal.pone.0232073)
Supplement: S3 Fig — The bold case base gives the location of rs76735376. Green background: POU2F1/POU5F1 binding site, Green font: mutated POU2F1/POU5F1 binding site, Blue CEBPB binding site. The reverse primers (-r) are given in reverse orientation, as they are annealed to the forward oligos (-f). (PDF) [file pone.0232073.s009.pdf]

```
Oligo2 LPA_EMSA2-C-f      5'-CATGGTGCAATCTTACATTTTCGTTCTCAT-3'
Oligo4 LPA_EMSA2-T-f      5'-CATGGTGCAATCTTACATTTTCATTCTCAT-3'
Oligo6 LPA_EMSA2-5mC-f    5'-CATGGTGCAATCTTACATTTTCGTTCTCAT -3'

Oligo2 LPA_EMSA2-C-r      3'-GTACCACGTTAGAATGTAAAAG CAAGAGTA-5'
Oligo4 LPA_EMSA2-T-r      3'-GTACCACGTTAGAATGTAAAAG TAAGAGTA-5'
Oligo6 LPA_EMSA2-5mC-r    3'-GTACCACGTTAGAATGTAAAAGmCAAGAGTA-5'
```

S3 Fig: Oligos used for EMSA experiments. The bold case base gives the location of rs76735376. Green background: POU2F1/POU5F1 binding site, Green font: mutated POU2F1/POU5F1 binding site, Blue CEBPB binding site. The reverse primers (-r) are given in reverse orientation, as they are annealed to the forward oligos (-f).
