# Supplementary material for: A genome-wide analysis of DNA methylation identifies a novel association signal for Lp(a) concentrations in the LPA promoter
Source: PLoS One. 2020 Apr 28;15(4):e0232073. doi: 10.1371/journal.pone.0232073 (PMC7188291; doi:10.1371/journal.pone.0232073)
Supplement: S4 Fig — (PDF) [file pone.0232073.s010.pdf]

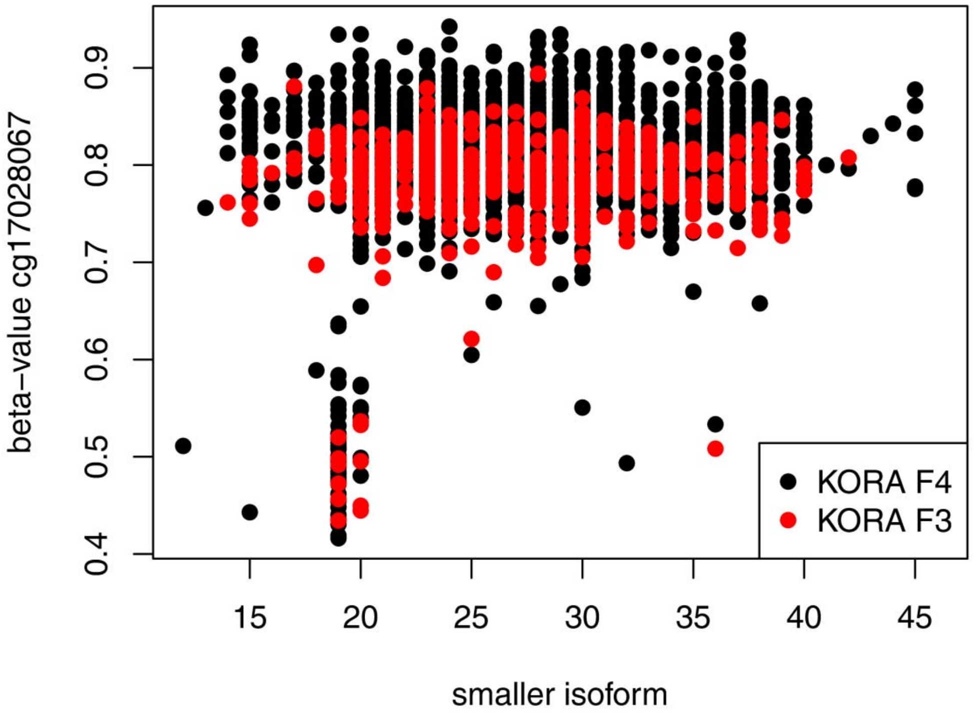

S4 Fig: Scatterplot showing the lower of both apo(a) isoforms (x-axis) per person in KORA F3 (in red) and KORA F4 (in black) versus the beta-value of cg17028067 (y-axis)
