# Supplementary material for: A genome-wide analysis of DNA methylation identifies a novel association signal for Lp(a) concentrations in the LPA promoter
Source: PLoS One. 2020 Apr 28;15(4):e0232073. doi: 10.1371/journal.pone.0232073 (PMC7188291; doi:10.1371/journal.pone.0232073)
Supplement: S5 Fig — Panel A: Representative results of bisulfite sequencing in two homozygotes for the major allele and two heterozygotes in the SAPHIR study. The blue peak represents the unconverted C-allele, indicating that the major part is methylated in CC carriers, whereas only a minor part is methylated in CT carriers. Panel B: Boxplots of the methylation level (expressed as beta-value) of cg17028067, stratified for genotypes in the KORAF4 study (panel B). (PDF) [file pone.0232073.s011.pdf]

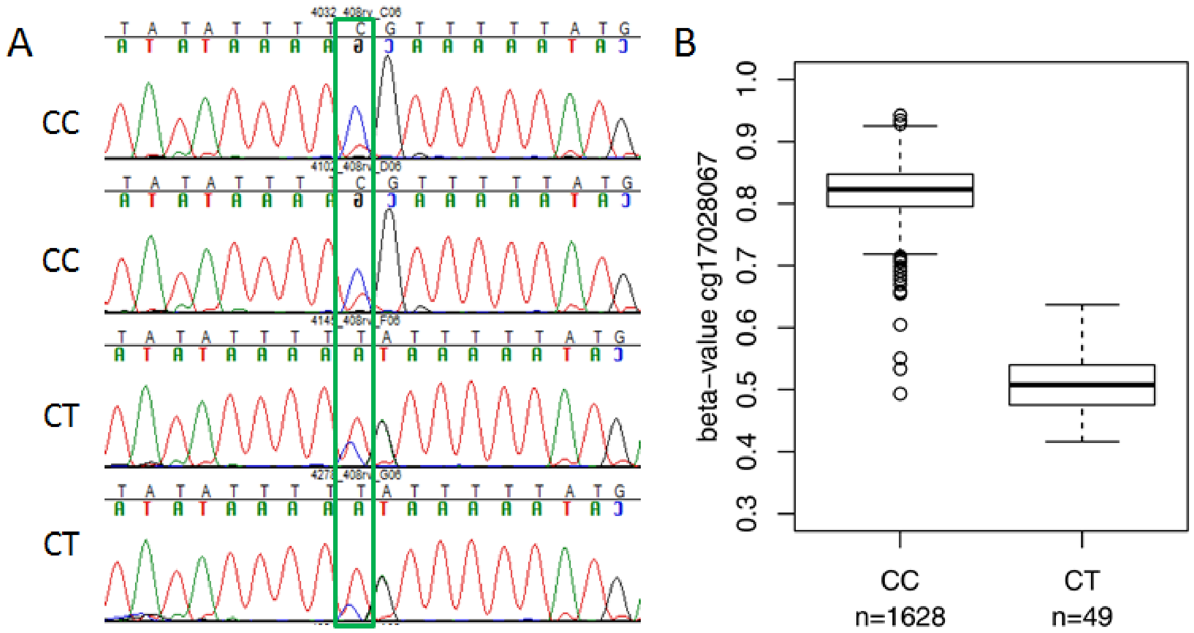

S5 Fig: Panel A: Representative results of bisulfite sequencing in two homozygotes for the major allele and two heterozygotes in the SAPHIR study. The blue peak represents the unconverted C-allele, indicating that the major part is methylated in CC carriers, whereas only a minor part is methylated in CT carriers. **Panel B:** Boxplots of the methylation level (expressed as beta-value) of cg17028067, stratified for genotypes in the KORAF4 study (panel B).
