# Supplementary material for: A genome-wide analysis of DNA methylation identifies a novel association signal for Lp(a) concentrations in the LPA promoter
Source: PLoS One. 2020 Apr 28;15(4):e0232073. doi: 10.1371/journal.pone.0232073 (PMC7188291; doi:10.1371/journal.pone.0232073)
Supplement: S6 Fig — Distribution of the smaller of both isoforms, stratified for carriers (CT or TT) and non-carriers (CC) of the SNP rs76735376 rs76735376 (panel A) and carriers and non-carriers of rs10455872 (panel B) (PDF) [file pone.0232073.s012.pdf]

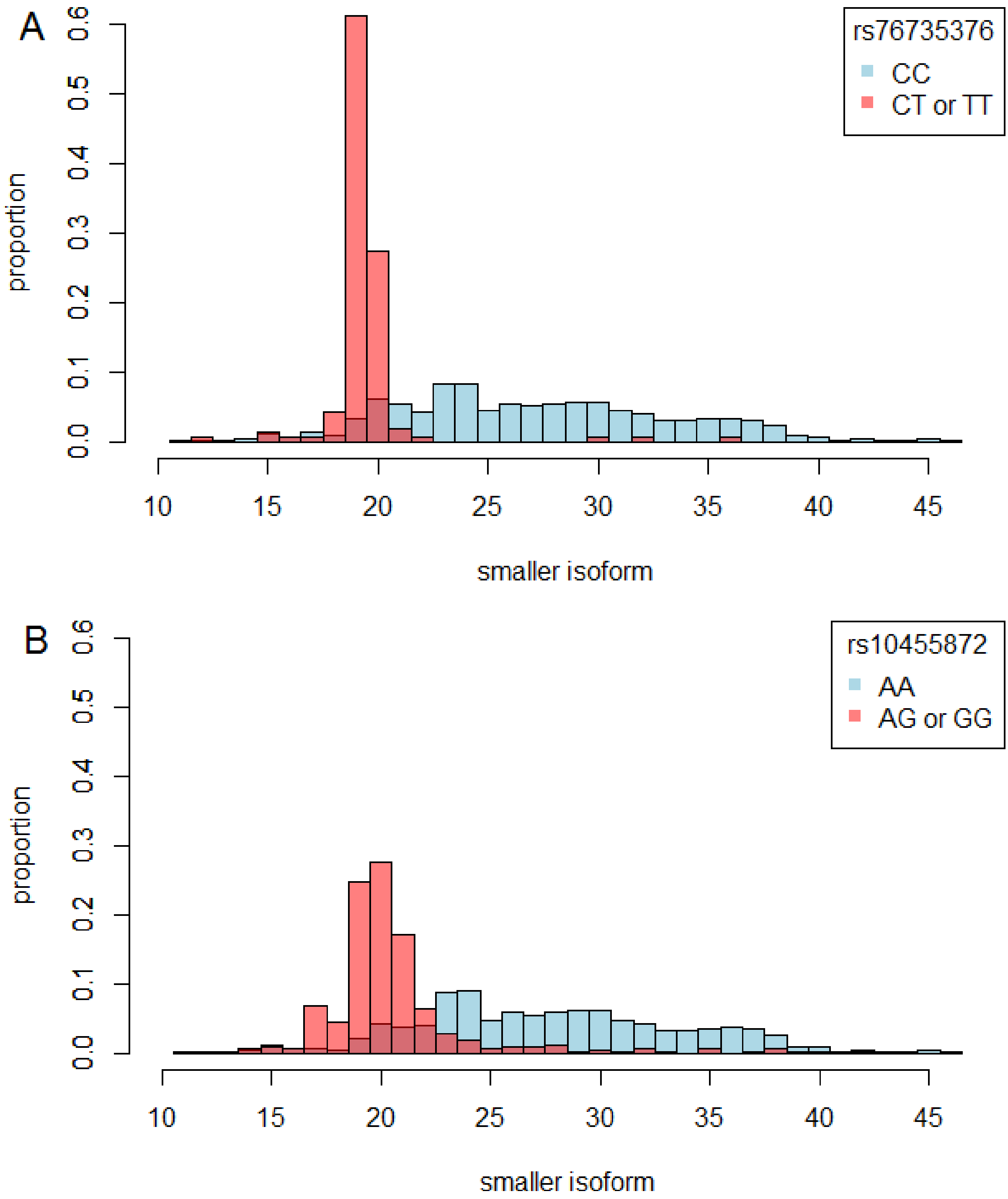

S6 Fig: Distribution of the smaller of both isoforms, stratified for carriers (CT or TT) and non-carriers (CC) of the SNP rs76735376 (panel A) and carriers and non-carriers of rs10455872 (panel B)
