# Supplementary material for: A genome-wide analysis of DNA methylation identifies a novel association signal for Lp(a) concentrations in the LPA promoter
Source: PLoS One. 2020 Apr 28;15(4):e0232073. doi: 10.1371/journal.pone.0232073 (PMC7188291; doi:10.1371/journal.pone.0232073)
Supplement: S8 Fig — rs76735376 modifies binding of the transcription factor CEBPB. Left panel: Diagram of the double-stranded oligos used for EMSA analysis containing either cytosine (red, Oligo2 LPA_EMSA2-C), thymine (Oligo4 LPA_EMSA2-T) or methyl-cytosine (6 LPA_EMSA2-5mC) oligo. The transcription factor binding sites for CEBPB (blue) and POU2F1/POU5F1 (green) as predicted by Transfac analysis, 52 of the rs76735376 probes are shown. Position weight matrix of the TF is shown. The arrow indicates the orientation of the sequence matrix in the oligo. Right panel: EMSA analysis of human liver nuclear extract hybridized to Oligo2 LPA_EMSA2-C, Oligo4 LPA_EMSA2-T or Oligo6 LPA_EMSA2-5mC. Binding specificity to specific transcription factors was performed by using super-shift antibodies (s-shift AB) for the POU factors or CEBPB. An NFATC1 antibody and excess of cold unlabeled oligonucleotide (cold comp.) were used as negative control. One representative experiment out of three is shown. Note that the free probe has already left the gel because a long run time in order was required to distinguish the super-shift bands. (PDF) [file pone.0232073.s014.pdf]

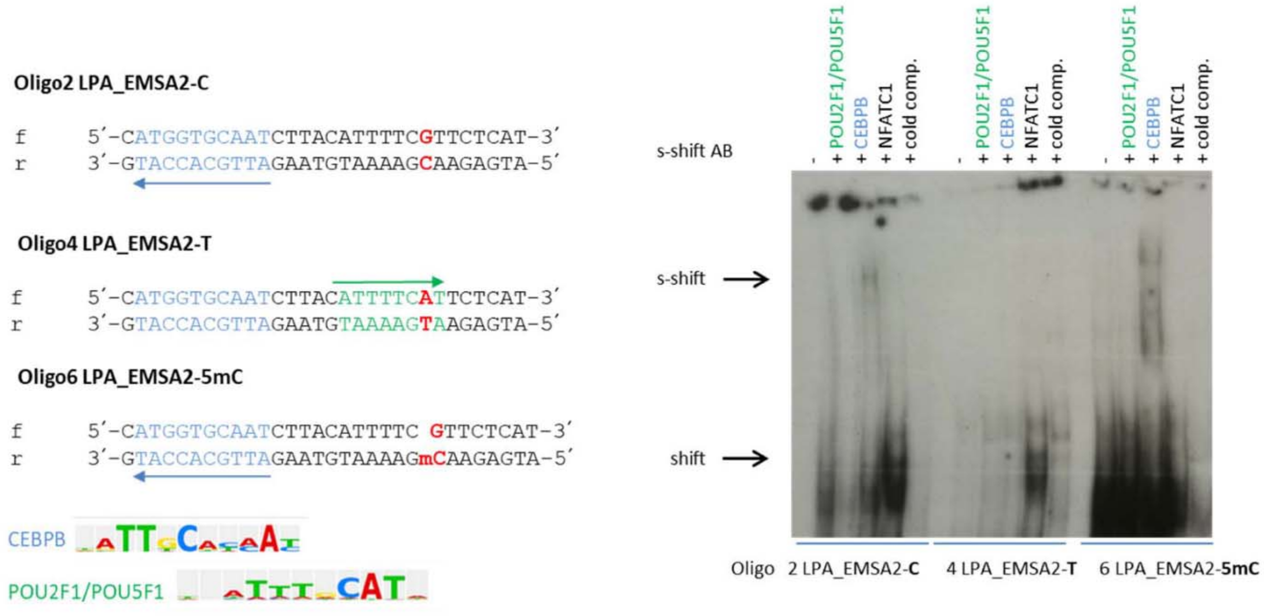

S8 Fig: Electrophoretic mobility shift assay for rs76735376

rs76735376 modifies binding of the transcription factor CEBPB. Left panel: Diagram of the double-stranded oligos used for EMSA analysis containing either cytosine (red, Oligo2 LPA_EMSA2-C), thymine (Oligo4 LPA_EMSA2-T) or methyl-cytosine (6 LPA_EMSA2-5mC) oligo. The transcription factor binding sites for CEBPB (blue) and POU2F1/POU5F1 (green) as predicted by Transfac analysis, 52 of the rs76735376 probes are shown. Position weight matrix of the TF is shown. The arrow indicates the orientation of the sequence matrix in the oligo. Right panel: EMSA analysis of human liver nuclear extract hybridized to Oligo2 LPA_EMSA2-C, Oligo4 LPA_EMSA2-T or Oligo6 LPA_EMSA2-5mC. Binding specificity to specific transcription factors was performed by using super-shift antibodies (s-shift AB) for the POU factors or CEBPB. An NFATC1 antibody and excess of cold unlabeled oligonucleotide (cold comp.) were used as negative control. One representative experiment out of three is shown.

Note that the free probe has already left the gel because a long run time in order was required to distinguish the super-shift bands.
