# Supplementary material for: A genome-wide analysis of DNA methylation identifies a novel association signal for Lp(a) concentrations in the LPA promoter
Source: PLoS One. 2020 Apr 28;15(4):e0232073. doi: 10.1371/journal.pone.0232073 (PMC7188291; doi:10.1371/journal.pone.0232073)
Supplement: S9 Fig — The x-axis shows p-values from a GWAS on Lp(a) (using data from the KORA F4 study), the y-axis p-values from eQTL analysis (GTEx consortium V8). The index SNP (rs76735376) is marked as purple triangle. Only those SNPs are shown, which show a r2 of ≥ 0.1 with the index SNP (LD information derived from 1000 genomes CEU). Color coding shows magnitude of LD. (PDF) [file pone.0232073.s015.pdf]

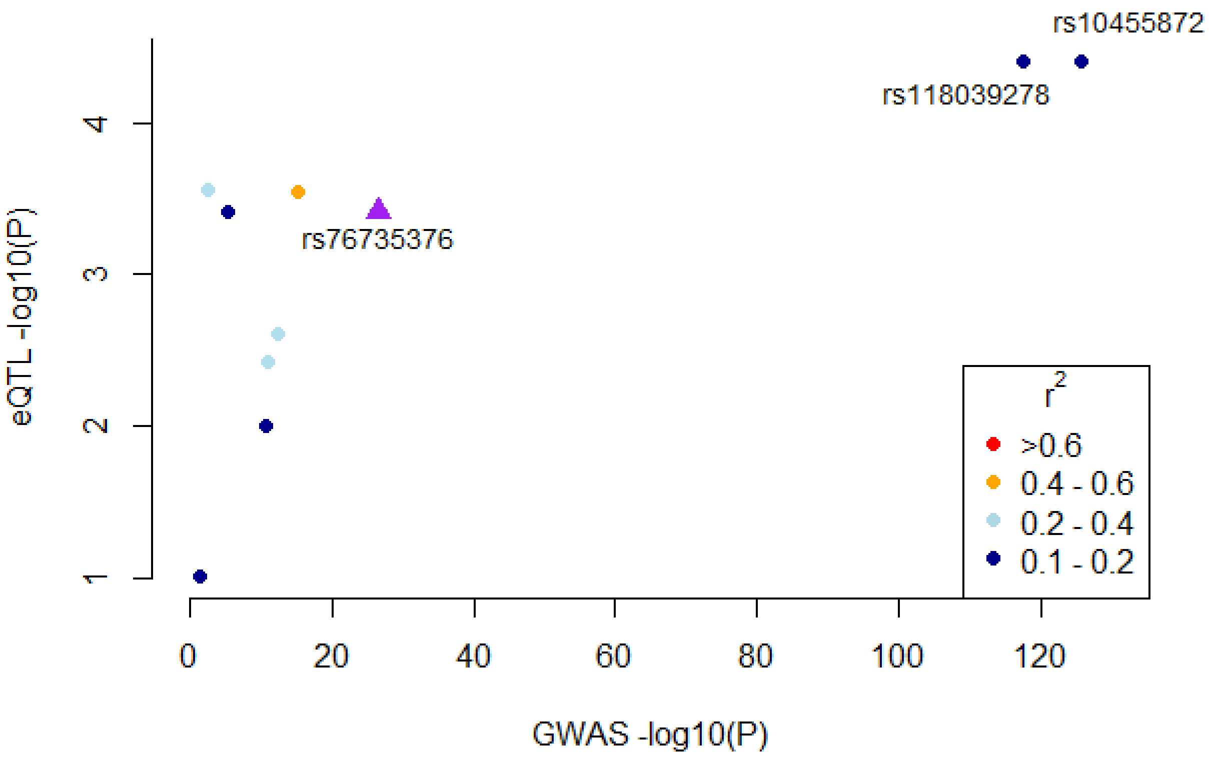

S9 Fig: Scatterplot of p-values from a GWAS on Lp(a) versus p-values from eQTL analysis

The x-axis shows p-values from a GWAS on Lp(a) (using data from the KORA F4 study), the y-axis p-values from eQTL analysis (GTEx consortium V8). The index SNP (rs76735376) is marked as purple triangle. Only those SNPs are shown, which show a r^2 of ≥ 0.1 with the index SNP (LD information derived from 1000 genomes CEU). Color coding shows magnitude of LD.
