# Supplementary material for: A genome-wide analysis of DNA methylation identifies a novel association signal for Lp(a) concentrations in the LPA promoter
Source: PLoS One. 2020 Apr 28;15(4):e0232073. doi: 10.1371/journal.pone.0232073 (PMC7188291; doi:10.1371/journal.pone.0232073)
Supplement: S2 Fig — Upper case: sequence regions, which could be covered with amplicons for bisulfite sequencing. Lower case: regions which could not be covered by amplicons for bisulfite sequencing. Blue background (light and dark): primer binding sites for bisulfite sequencing. Note that the sequence given here is the unconverted sequence. The primer given in S2 Table bind on the converted sequence and the complement strand of the strand shown here. Yellow background: primer binding sites of the sequences used for SNP validation by sequencing. Bold character: LPA pentanucleotide repeat. Green: CpG affected by rs76735376 (enlarged cytosine base). Position of rs76735376 is underlined. Pink: CpG in the region, which were amenable to sequencing. Purple: CpG in the region, which were not amenable to sequencing (not represented in any amplicon). Sequence is the bisulfite-converted sequence given 5’ to 3’ on the minus strand of human genome GRCh37/hg19 (i.e. the same direction as LPA transcription direction). (PDF) [file pone.0232073.s008.pdf]

```
ctatgagtttctgaatggaagagtcaaatccactgagctctgtgctgggcatttggctggaggcattg
acagttgcaaggtaagaaaagatcaagagaccaaagttagtcttgtgctctcctgtctcagtctcagt
cccttagacttgagtcccaaagtagcgaattcaagtaggatttaatcaatggaagaccccagtctaag
tgttgctcagaaactccctagatctgtcccaaatgtatattcagatcatccaaggggacttcttgggg
cttgagttccagatcagcagcaagggagccataagtgccataactacctcAGACCACTCACCCTCCTG
GGGTGTCCCGGTGGCCAGGGACTAAAGTGGTGATTTTTCTGGTAGGGAAGGAGGTAGAGGGTACAGGA
CAGAGACTAACTGCACACAATATCTGAGACTGGAGCTCAGATATTGCTGATGATCAGAGTTGGCGTGT
CTCCCCAATTGATTTACAACTGGGGCTTGGATACTGTTTTAAACGGGAGGAGCCTCCTAACCATCTTG
ACACAACCACTGACGTGACTACACTAGAATAGACTCTTTCCACTTAATTCTACCACTCTTGCTTTACT
TCATGAGAACGAAAATGTAAGATTGCACCATGAATTCATTTGCGGAAAGATTGATACTATGCTTTTAT
TTTATTTTATTTTATTTTATTTTATTTTATTTTATTTTATTGAGACTCTCACCCCGGTTGAAGTGCAC
TGACGTGATTTTGGCTCACTGCAACTTCCACCTCCTgggttcaagtgaatactccagcctccctagta
gctgggattacaggtgcccaccaccacgcctggctaattttgtatttttagtagagatggggtttca
ccacattggcctggctggtctcaaactcctgaccttgtgatccacctgtcttggcctcccaaagtgct
gggattacagagttgagccaccgcactcgaccctatgtttatttttaaaaatatttatttatttatt
taagccacaactactagaataggaaggattgatattttattaattttatttggtatttattattttt
tttctttcctgagacattcttgctctgtcacccaggctggagtgcagtggcacattcttggctcactg
caacctccatctcctgtgttcaagcaattctagtgcctcagcctacttagtagcTGGGATGACTGGCA
TGTGCCTCCACACCCAGCTAATTTTTGTATTTTTTGTAGAGACAGGGTTTTGGCATGTTGCCCAGGCT
TGTCTCAAACTCCTGGCCTCAGGTGATCCATCTGCCGTGGCCTCCCAAAATGCTGGGATTATAGGCAT
GAGCCACCACCCCCTCCTGGAAGGATTGATATCTTATAACATAATTTATAATTACAGAAAACATGTGA
GTTCACTAGGAATAAATAAATTTTGAAGATAATAAAAGATTTTCACTTATGTTGTCATTTCGGCACAG
TTTGGTATAGGATGTGGAGATGTTAACATTTATACCTAGCTTGCTCGTAAACTAAGACCTGAAAGGGT
TGTGTCTATCAGCTGCACCCCTGGGTAGCGACACAACCTCGGGAAGGCCTCAGCCCCCTCCTCGTACA
GCACTGCCTGTTGGAAAGCTTGAGGGAGGCTATGGATGTGCAGCACTTGGCAGAGGGTCTGGtcatgg
aagttaccagcaaatatgagctactttatgattttattttatccaaaagaaagagaatgaaagaaga
ggggaggaaacaagactaatcaggaaagatgaaggtctaggggtgagggaaggagtaaggagacataa
aggcaatgtggagcagctgagggggaaatggctttcaccacttcccagcatctattgacattgcact
ctcaaatattttataagactctatattcaaggtaatgtttgaaccctgctgagccagtggcatgggtc
tctgagagaatcattaacttaatttgactatctggtttgtgggtgcgtttactctcatgtaagtcaa
```

S2 Fig: Overview of the region around rs76735376 with location of the LPA pentanucleotide repeat, the location of the amplicons, the location of the CpG affected by rs76735376 and neighboring CpG sites.

Upper case: sequence regions, which could be covered with amplicons for bisulfite sequencing. Lower case: regions which could not be covered by amplicons for bisulfite sequencing.

Blue background (light and dark): primer binding sites for bisulfite sequencing. Note that the sequence given here is the unconverted sequence. The primer given in Supplementary Table 2 bind on the converted sequence and the complement strand of the strand shown here. Yellow background: primer binding sites of the sequences used for SNP validation by sequencing. Bold character: *LPA* pentanucleotide repeat. Green: CpG affected by rs76735376 (enlarged cytosine base). Position of rs76735376 is underlined. Pink: CpG in the region, which were amenable to sequencing. Purple: CpG in the region, which were not amenable to sequencing (not represented in any amplicon).

Sequence is the bisulfite-converted sequence given 5’ to 3’ on the minus strand of human genome GRCh37/hg19 (i.e. the same direction as LPA transcription direction).
